# Prognostic factors and outcomes in pediatric acute myeloid leukemia: a comprehensive bibliometric analysis of global research trends

**DOI:** 10.3389/fonc.2025.1466818

**Published:** 2025-02-17

**Authors:** Mingliang Rao, Wenna Luo, Caiju Luo, Baojing Wu, Tiantian Xu, Ziqian Wei, Haolan Deng, Kejing Li, Dunhua Zhou

**Affiliations:** ^1^ Children’s Medical Center, Sun Yat-sen Memorial Hospital, Sun Yat-sen University, Guangzhou, China; ^2^ Guangdong Provincial Key Laboratory of Malignant Tumor Epigenetics and Gene Regulation, Sun Yat-sen Memorial Hospital, Sun Yat-sen University, Guangzhou, China; ^3^ Department of Laboratory Medicine, Heyuan People’s Hospital, Heyuan, China

**Keywords:** pediatric AML, prognosis, bibliometric analysis, genetic markers, immune therapy

## Abstract

**Background:**

Pediatric AML prognosis research has advanced significantly, yet gaps in understanding genetic and molecular interactions persist. Despite improved outcomes, relapse/refractory cases and personalized treatment integration remain critical clinical challenges.

**Objective:**

To analyze the global research landscape on pediatric AML prognosis, highlight influential components and collaborations, and identify major potential research trends.

**Methods:**

Publications on pediatric AML prognosis research from 1999 to 2023 were retrieved from the Clarivate Analytics Web of Science Core Collection (WoSCC) database. Bibliometric analysis was conducted using CiteSpace and VOSviewer to identify leading countries, prominent institutions, high-impact journals, key research categories, influential authors, and emerging research topics.

**Results:**

The bibliometric analysis encompassed 924 publications, with St. Jude Children’s Research Hospital emerging as the most prolific institution. The United States leads globally in terms of countries, institutions, journals, and authors. Todd A. Alonzo ranks highest in publication volume, while U. Creutzig leads in citations. The top research categories were Oncology, Hematology, and Pediatrics. Key research topics included genomics, transcriptomics, epigenomics, targeted therapies, immune therapy, and integrative diagnostic approaches.

**Conclusion:**

This bibliometric analysis highlights significant advancements in pediatric AML prognosis over the past 25 years, driven by the integration of genetic markers, immunological insights, transcriptomics, and epigenomics, which have collectively transformed risk stratification and treatment strategies. Overcoming challenges, such as discovering new therapeutic targets and enhancing treatment combinations, will depend on global collaboration and advanced technologies to propel the field forward.

## 1 Introduction

Pediatric acute myeloid leukemia (AML) is a malignant hematological disorder characterized by the clonal proliferation of myeloid precursors in the bone marrow (1). Despite significant advances in the understanding and treatment of pediatric AML, it remains a leading cause of cancer-related mortality in children (2). Over the past few decades, considerable progress has been made in identifying genetic and molecular markers that play crucial roles in predicting prognosis and guiding treatment outcomes (3–6). These advancements have led to the development of targeted therapies and more precise risk stratification methods, significantly improving patient outcomes (7–9). However, high rates of relapse and refractory cases continue to underscore the need for ongoing research and innovation in this field (10).

A critical challenge in pediatric AML research is the disease’s inherent heterogeneity, which complicates both treatment decisions and prognosis (11, 12). While key genetic mutations and molecular abnormalities—such as FLT3-ITD, WT1, CEBPA mutations, and KMT2A rearrangements—have been identified as important prognostic markers (13–16), there remain significant gaps in our understanding of how these markers interact and contribute to disease progression and therapeutic resistance (17). Addressing these gaps is essential to overcoming the persistent challenges of relapse and refractory disease, and to better integrating personalized treatment strategies into clinical practice.

This analysis provides a comprehensive bibliometric overview of global research trends in pediatric AML prognosis from 1999 to 2023. By examining key aspects such as publication trends, leading countries, prestigious institutions, influential journals, major research categories, notable contributors, seminal references, and emerging trends, it aims to offer a clear snapshot of the current landscape and future directions of the field. The analysis specifically focuses on how advancements in genomics, transcriptomics, and epigenomics have shaped prognosis and treatment outcomes. Furthermore, it explores the pivotal role of clinical trials in developing treatment protocols and highlights emerging therapies that show promise for improving patient outcomes.

This bibliometric approach synthesizes extensive research data, providing a detailed and systematic overview of the field. By identifying significant trends and key studies, it offers valuable insights for researchers, clinicians, and policymakers alike. The findings are expected to contribute to the development of more effective, personalized treatment strategies, ultimately improving the prognosis for pediatric AML patients. Notably, this is the first bibliometric study to comprehensively explore prognosis-related research in pediatric AML, marking a significant contribution to the field.

## 2 Data sources and search methodology

We utilized the Clarivate Analytics Web of Science Core Collection database (WoSCC) to identify and gather publications on prognosis-related pediatric AML research. The search algorithm used was as follows: Topic search #1 included “pediatric acute myeloid leukemia”,”childhood acute myeloid leukemia”, “children acute myeloid leukemia,”,”pediatric AML”, “childhood AML”, “children AML” and Topic search #2 included “prognosis”, “outcome”, “survival”, “predictive factors”, “risk factors”, “long-term outcomes”, “prognostic factors”, “treatment response”, “relapse” and “remission” with a publication date range of 1999-2023. This search yielded 1160 articles in the WoSCC database.

**Figure 1:**
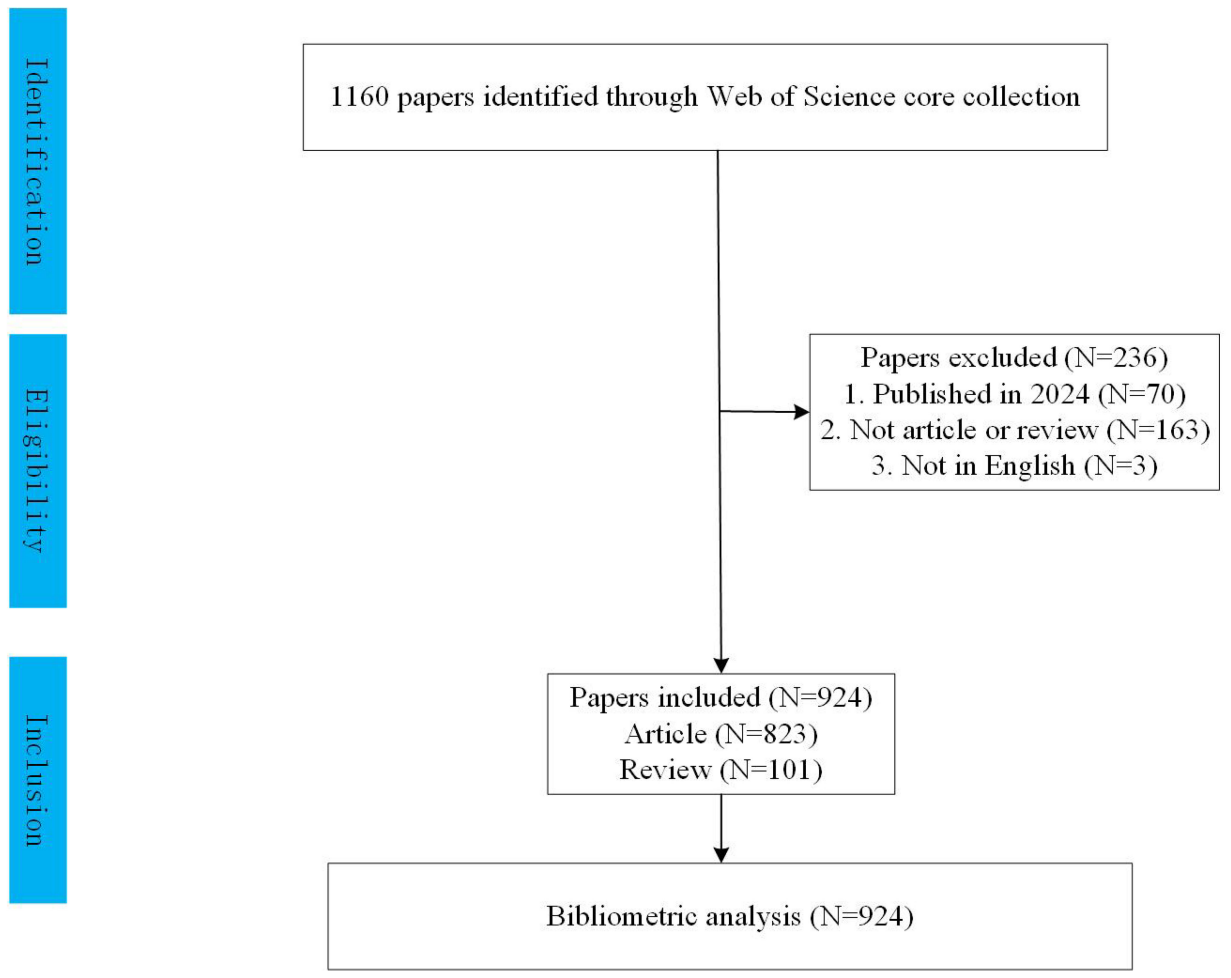
Flow chart of the publications extracted from the database.

We excluded conference abstracts, editorials, and letters to focus on original research and reviews. To minimize bias, two independent researchers conducted the search together, completing it on February 3, 2024. Titles and abstracts were manually reviewed to select studies specifically addressing prognosis in pediatric AML. Irrelevant articles or those offering limited insights were excluded. We further refined our selection to include only English-language articles and reviews within the specified date range, resulting in 924 publications for analysis, as shown in **Figure 1**. All records were then imported into CiteSpace v.5.8.R2.

This study analyzed publicly available bibliometric data on pediatric AML prognosis, focusing on published titles and abstracts, with no involvement of human participants or primary data collection. Ethical approval was not required, as the study adhered to international guidelines for bibliometric research, which do not necessitate ethical review for publicly available data without personal identifiers. We followed established ethical standards for data handling, including proper citation and transparency, in line with frameworks from the ISSI and OECD Guidelines for the Responsible Use of Bibliometrics.

### 2.1 Tools for bibliometric analysis

For our bibliometric analyses, we employed Citespace (version 6.3.R2) and VOSviewer (version 1.6.20).

### 2.2 Data visualization and presentation

In our bibliometric analysis, we applied several mathematical techniques to uncover significant patterns and trends. Key methods included frequency and co-occurrence analysis, which identified the most discussed topics and their interrelationships. We also used cluster analysis to group related articles, authors, or keywords into distinct clusters, revealing thematic concentrations and research activity patterns. Network analysis played a crucial role in mapping citation and co-authorship networks, highlighting influential authors and publications while visualizing the collaborative landscape. Temporal analysis tracked the evolution of research themes, allowing us to identify both persistent and emerging trends over time (18). For reader clarity, we have provided a brief tutorial on interpreting these visualizations. In CiteSpace, nodes represent articles, authors, or keywords, with size indicating citation count or frequency, while connecting lines show co-citations or co-occurrences (19). In VOSviewer, network diagrams highlight related groups, with proximity indicating stronger connections and colors representing distinct clusters.

Furthermore, We provide illustrative examples to demonstrate the practical application of our results. One network map highlights a key research cluster focused on FLT3 inhibitors and their impact on survival rates in high-risk pediatric AML patients, featuring significant publications and leading authors. Another example is our temporal analysis, which shows the shift in pediatric AML treatment from traditional chemotherapy to combined strategies involving chemotherapy, transplantation, immunotherapy, and targeted therapies. As treatment approaches have become more personalized, patient prognosis has significantly improved.

CiteSpace enabled the extraction of publication and citation graphs, country collaboration networks, and the 25 most prominent keywords based on citation bursts. VOSviewer was essential for visualizing collaborative networks among countries, journals, and authors, as well as conducting keyword analyses. Microsoft Excel 2019 (Microsoft, Redmond, WA, USA) was used to illustrate publication trends and predict next year’s output. The H-index of journals was calculated following Hirsch’s method, which ranks articles by citation count and identifies the number “H” where “H” articles have been cited at least “H” times (20).

## 3 Results

### 3.1 Annual trends in global publications

**Figure 2:**
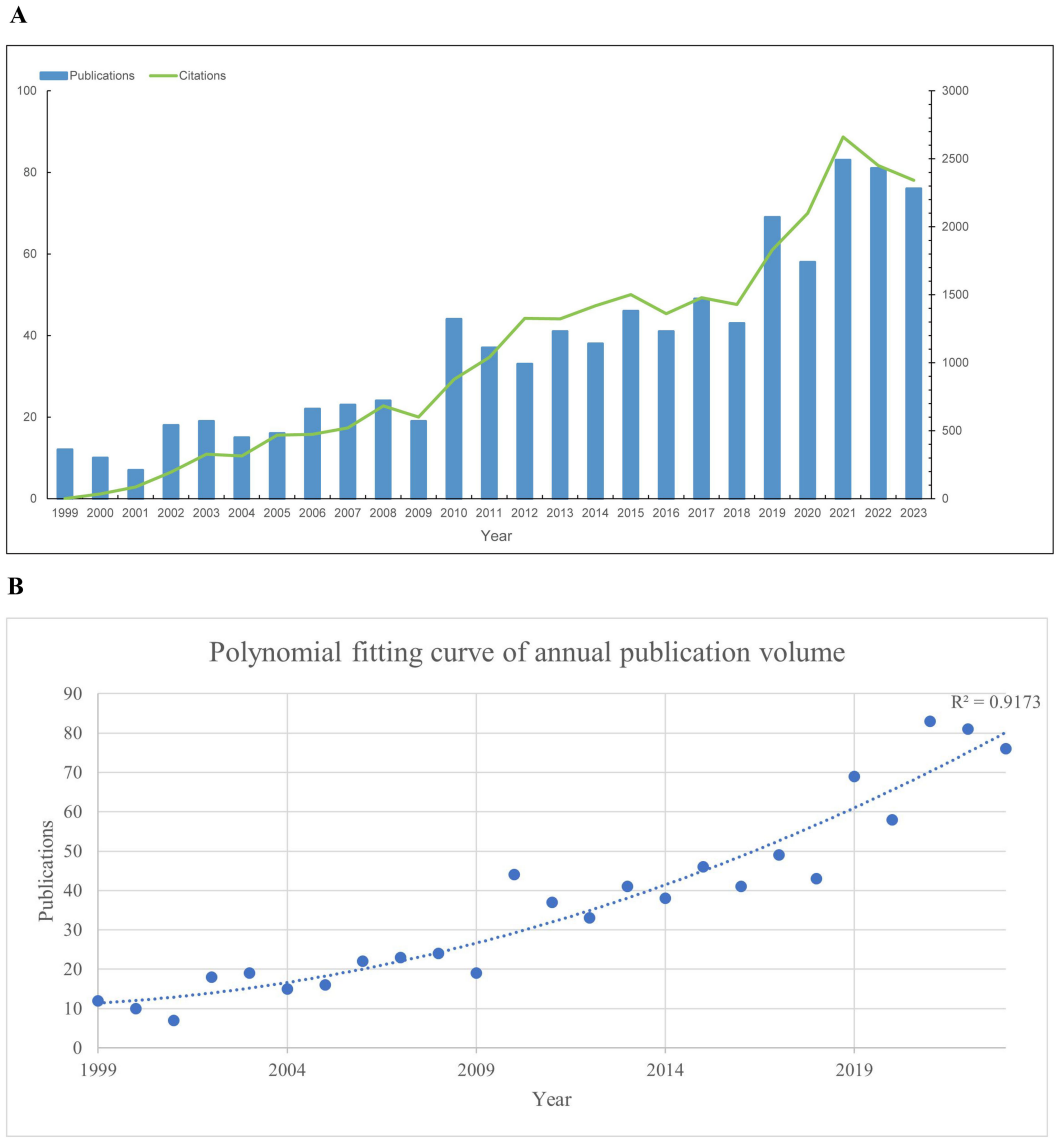
Annual trends in global publications and citations on prognosis-related pediatric AML literatures. **(A)** Annual trends in global publications and citations on prognosis-related pediatric acute myeloid leukemia (AML) research from 1999 to 2023. **(B)** Polynomial fitting curve of annual trends in global publications.

Our bibliometric analysis incorporated 924 publications from 1999 to 2023, with 89% being original research articles and 11% comprehensive reviews. The analysis reveals a steady increase in the number of publications and citations related to prognosis in pediatric AML research from 1999 to 2021, reaching a peak in 2021. Following this peak, a slight decline is observed from 2021 to 2023. Notably, the annual publication output doubled between 2013 and 2021 (**Figure 2A**). To elucidate this trend, a polynomial fitting curve was utilized. The analysis yielded a coefficient of determination (R²=0.9173), underscoring the statistical significance of the observed pattern (**Figure 2B**). These data highlight the growing interest and investment in prognosis-related pediatric acute myeloid leukemia (AML) research.

### 3.2 Countries and regions

**Figure 3:**
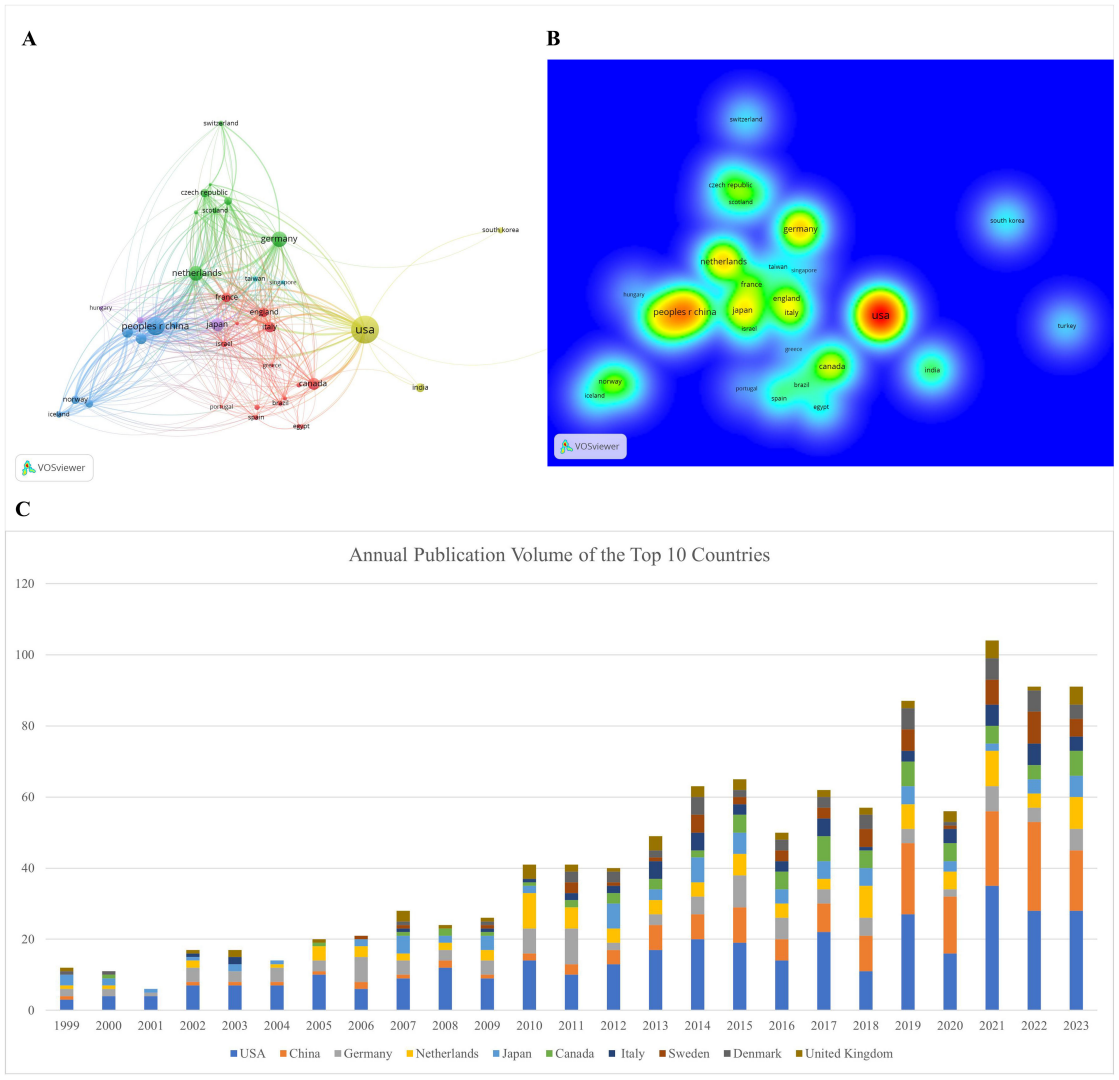
Visualization of international collaborations in prognosis-related pediatric AML research. **(A)** The graph illustrates collaborations between countries in prognosis-related pediatric AML research. The size of each bubble corresponds to the number of publications from each country, while the thickness of the connecting lines between nodes represents the strength of collaborative ties, indicating the extent of their collaborative efforts. Connection lines between countries show the cooperation relationships, highlighting the global network of research partnerships. **(B)** Density visualization of collaborations between countries. This visualization displays the density and intensity of collaborative research activities between countries in the field of pediatric AML prognosis. The heatmap is employed to highlight regions of varying collaborative strength, with warmer colors representing areas of higher activity and stronger connections. **(C)** Annual publication volume of the top 10 countries.

Investigators from 65 countries or regions have contributed to the study of prognosis-related pediatric AML. **Table 1** and **Figure 3C** highlight the top 10 most productive nations in this research field. Europe emerges as a dominant contributor with five countries, while North America and Asia each have two, and Oceania has one. The United States stands out prominently, having the highest number of publications, total citations, and an impressive H-index of 63, indicating both the quantity and quality of its academic output. Moreover, the network view and heatmap illustrate the USA as a major hub, represented by the brightest (red) color, demonstrating extensive collaborative links with numerous countries, with significant participation from North America and Europe (**Figures 3A, B**). These visualizations effectively identify significant international research networks and highlights areas for potential growth in collaboration, underscoring the United States’ leadership in the study of prognosis-related pediatric AML. Furthermore, Germany is distinguished by achieving the highest average citation per paper, reflecting the nation’s production of high-quality studies with significant influence. Notably, China, the only developing country among the top 10, has demonstrated remarkable collaboration in this field, with high-density collaborative links with surrounding countries (**Figure 3A**) and a prominent red area (**Figure 3B**).

**Table 1:** Top 10 productive countries/regions in pediatric AML prognosis research.

Rank	Country/regions	Count	Total citations	H-index	Average citation per paper
1	United States	352	14721	63	41.82
2	China	169	2308	27	13.82
3	Germany	111	6483	47	58.41
4	Netherlands	104	4976	39	47.85
5	Japan	82	3391	27	41.35
6	Canada	67	2429	26	36.25
7	Italy	55	2527	26	45.95
8	Sweden	54	1697	20	31.43
9	Denmark	52	2133	23	41.02
10	United Kingdom	49	2766	26	56.45

### 3.3 Institutions

**Figure 4:**
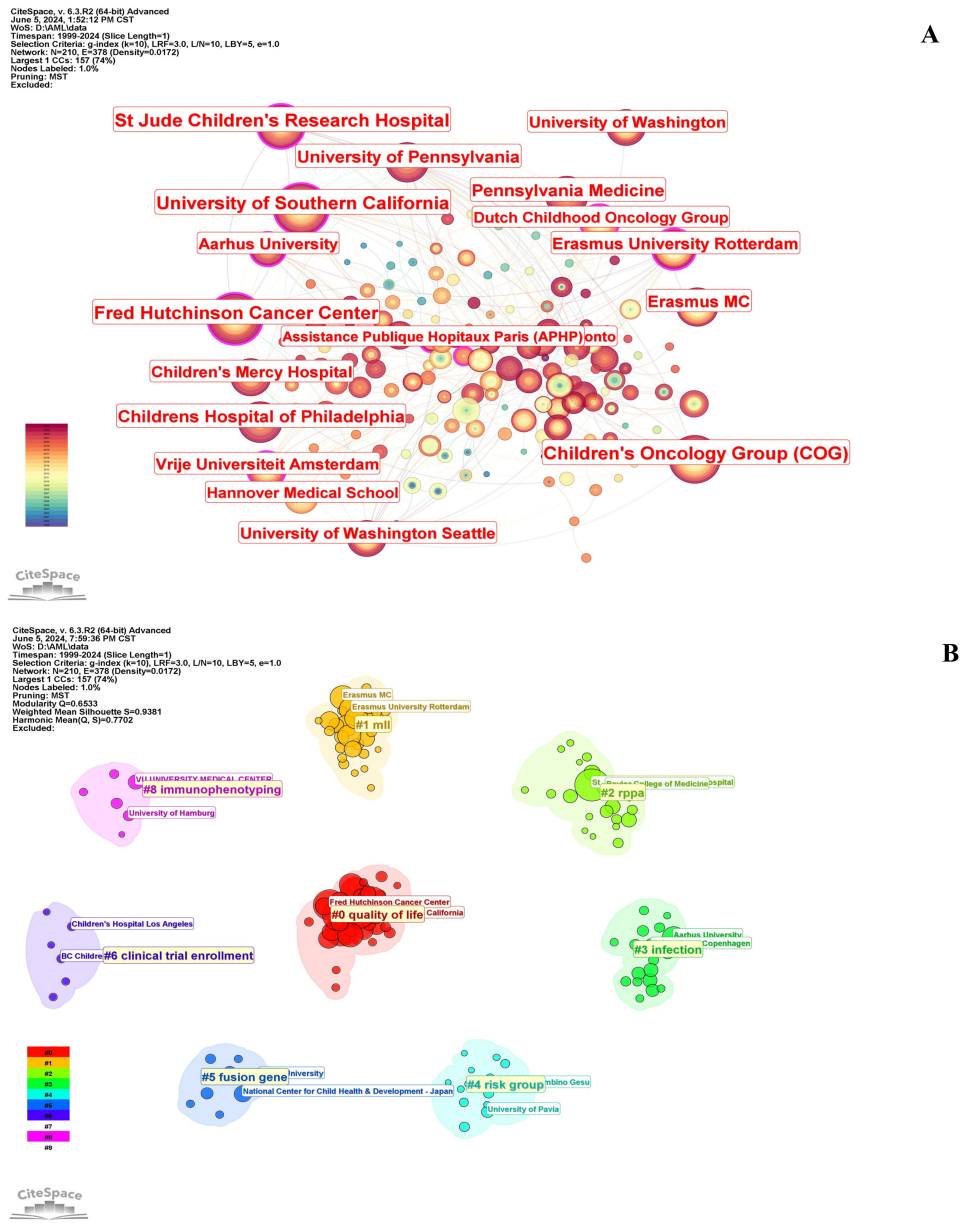
Collaborations between institutions in pediatric AML prognosis research. **(A)** Visualization of collaborations between institutions in pediatric AML prognosis research with a minimum of 5 publications. The graph illustrates collaborations between institutes involved in prognosis-related pediatric AML research. Each node represents an institute, with the node size indicating the volume of their publications. The lines between the nodes represent collaborative relationships between the institutions. **(B)** Circular cluster view of institutions involved in pediatric AML prognosis research (LLR). This visualization presents a circular cluster view of institutions engaged in this field. Each cluster is color-coded and spatially organized to reflect the density and strength of research activities. This circular cluster view facilitates the identification of leading institutions and their collaborative networks, providing insights into the institutional landscape of pediatric AML prognosis research.

**Table 2** shows that the top 10 institutions in pediatric AML prognosis research are mainly from North America and Europe, with eight based in the U.S. and two in the Netherlands. St. Jude Children’s Research Hospital is the most prolific, while Erasmus University Rotterdam leads in centrality (0.18), highlighting its key role in the research network. The Children’s Oncology Group (COG) has been pivotal in advancing pediatric AML prognosis through influential clinical trials and risk stratification strategies, contributing significantly to the field from 1999 to 2023. **Figure 4A** illustrates the strong institutional collaborations, primarily between U.S. and European institutions, emphasizing the global nature of this research. The density of connections in the figure reflects the frequency and strength of these partnerships, with COG acting as a central collaborative hub, linking with institutions like the University of Southern California and St. Jude Children’s Research Hospital.

**Table 2:** Leading institutions in pediatric AML prognosis research.

Rank	Institutions	Countries/regions	Count	Centrality
1	St Jude Children's Research Hospital	United States	105	0.16
2	University of Southern California	United States	97	0.14
3	Fred Hutchinson Cancer Center	United States	95	0.14
4	Children's Oncology Group (COG)	United States	91	0.04
5	Children’s Hospital of Philadelphia	United States	76	0.00
6	University of Pennsylvania	United States	74	0.08
7	Pennsylvania Medicine	United States	72	0.00
8	Erasmus MC	Netherlands	55	0.01
9	Erasmus University Rotterdam	Netherlands	55	0.18
10	Children's Mercy Hospital	United States	53	0.05

**Figure 4B** depicts a circular cluster view categorizing institutions into eight distinct research clusters, each represented by a color and corresponding to specific pediatric AML prognosis themes, highlighting the thematic diversity of institutional research and their collaborative networks. For example, Cluster #5 (blue) focuses on fusion genes, with notable contributions from the National Center for Child Health and Development in Japan. Similarly, Cluster #8 (pink) emphasizes immunophenotyping, led by institutions such as the University Medical Center Hamburg.

### 3.4 Leading journals, co-cited journals and categories

**Figure 5:**
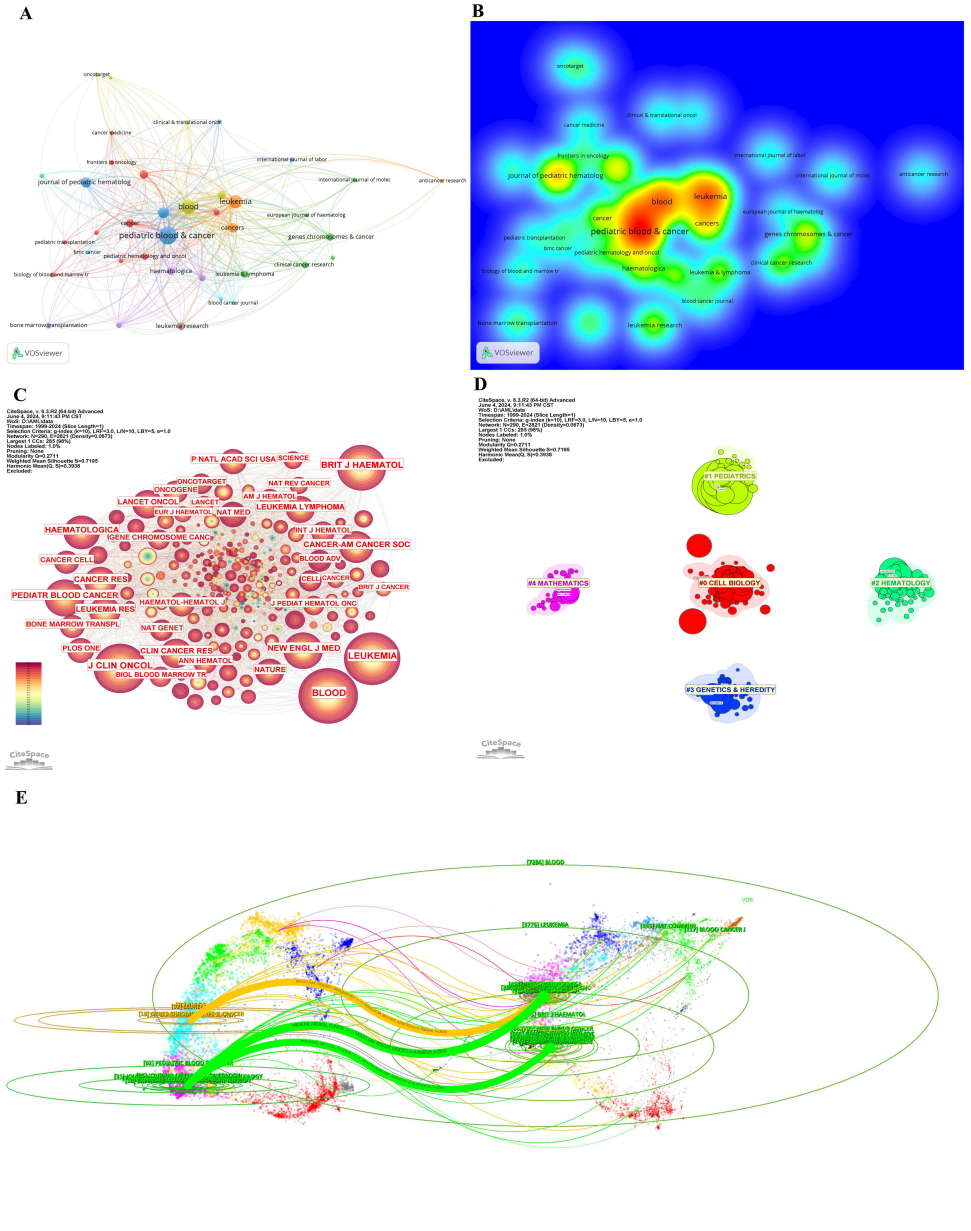
Journals and categories in pediatric AML prognosis research. **(A)** Visualization of collaborations between journals in pediatric prognosis AML research with a minimum of 5 publications. The graph illustrates collaborations between journals in prognosis-related pediatric AML research. Each node represents a journal, with the size of the node indicating the volume of their publications. The lines between nodes represent the collaborative relationships between the journals, while the thickness of the connecting lines between nodes represents the strength of collaborative ties. **(B)** Density visualization of journals featuring co-authorship. **(C)** The cited journals view in pediatric prognosis AML research. The graph illustrates collaborations between cited journals in this field. Each node represents a journal, with the node size indicating the citation count. The lines between the nodes represent collaborative relationships between the cited ones. **(D)** Circular cluster view of WoSCC Categories in pediatric prognosis AML research (LLR). This visualization presents a circular cluster view of categories engaged in this field. Each cluster is color-coded and spatially organized to reflect the density and strength of research activities. This circular cluster view facilitates the identification of leading categories and their collaborative networks, providing insights into the categories landscape of pediatric AML prognosis. **(E)** Dual-map overlay of journals in pediatric AML prognosis research. This dual-map overlay depicts the citation relationships between journals in the field of pediatric AML prognosis research. The base map shows citing journals on the left and cited journals on the right, with colored lines indicating citation pathways.

**Tables 3** and **4** present the top 10 journals by publication volume and citation impact in pediatric AML prognosis research. The leading co-cited and high-publication journals are primarily based in the United States. While publication volume and impact factor indicate a journal’s reach, citation count and H-index more accurately reflect its significance. Key contributors to the field include *Blood*, *New England Journal of Medicine (NEJM)*, *Lancet Oncology*, *Nature*, and *Nature Medicine*, which drive advancements in pediatric AML prognosis research (**Tables 3**, **4**, **Figures 5A–C**).

**Table 3:** Leading journals in pediatric AML prognosis research.

Rank	Journal title	Countries	Count	IF (2023)	JCR	Total citations	H index
1	Pediatric blood & cancer	United States	92	3.2	Q2	1867	25
2	Blood	United States	60	20.3	Q1	5846	43
3	Leukemia	England	52	11.4	Q1	3413	36
4	Journal of clinical oncology	United States	35	24.3	Q1	4109	30
5	Journal of pediatric hematology oncology	United States	31	1.2	Q4	155	8
6	Cancers	Switzerland	25	5.2	Q2	180	8
7	International journal of hematology	Japan	22	2.1	Q4	217	10
8	Cancer	United States	19	6.2	Q1	822	14
9	Blood advances	United States	19	7.5	Q1	302	9
10	Haematologica	Italy	18	10.1	Q1	477	11

**Table 4:** Leading cited journals in pediatric AML prognosis research.

Rank	High Cited Journal	Countries	Count	Centrality	Year
1	BLOOD	United States	871	0.02	1999
2	LEUKEMIA	United Kingdom	791	0.02	1999
3	J CLIN ONCOL	United States	724	0.01	1999
4	BRIT J HAEMATOL	United Kingdom	612	0.01	1999
5	NEW ENGL J MED	United States	395	0.04	1999
6	PEDIATR BLOOD CANCER	United States	382	0.01	2007
7	HAEMATOLOGICA	Italy	346	0.01	2002
8	CANCER-AM CANCER SOC	United States	329	0.03	1999
9	CANCER RES	United States	293	0.04	1999
10	LEUKEMIA RES	United Kingdom	263	0.05	1999
11	CLIN CANCER RES	United States	259	0.07	2002
12	LEUKEMIA LYMPHOMA	United Kingdom	246	0.07	1999
13	P NATL ACAD SCI USA	United States	238	0.11	1999
14	LANCET ONCOL	United Kingdom	223	0.01	2004
15	NATURE	United Kingdom	217	0.05	1999
16	CANCER CELL	United States	182	0.05	2003
17	ONCOGENE	United Kingdom	177	0.05	1999
18	NAT MED	United States	177	0.07	2002
19	BONE MARROW TRANSPL	United Kingdom	174	0.07	1999
20	PLOS ONE	United States	172	0.04	2013

**Figures 5A, B** visualize the collaboration networks and density of journal relationships, highlighting *Blood* as the most influential journal, with strong collaborative ties denoted by warm colors. **Figure 5C** further illustrates connections among the most cited journals, with *Blood*, *Nature*, *NEJM*, *Journal of Clinical Oncology*, and *Leukemia* emerging as central hubs due to their high citation counts and dense collaborations.

**Figure 5D** categorizes journals into five clusters—Pediatrics, Mathematics, Hematology, Genetics and Heredity, and Cell Biology—each represented by a distinct color corresponding to specific WoSCC categories. These clusters reflect thematic concentrations within pediatric AML prognosis research. **Table 5** identifies Oncology, Hematology, and Pediatrics as the top three research categories, with citation counts and centrality measures providing insights into their relative influence.

**Table 5:** Leading WoSCC categories in pediatric AML prognosis research.

Rank	WOS Categories	Count	Centrality	Year
1	ONCOLOGY	507	0.48	1999
2	HEMATOLOGY	449	0.13	1999
3	PEDIATRICS	188	0.23	1999
4	GENETICS & HEREDITY	47	0.14	1999
5	BIOCHEMISTRY & MOLECULAR BIOLOGY	32	0.23	2007
6	IMMUNOLOGY	31	0.08	2003
7	MEDICINE, RESEARCH & EXPERIMENTAL	27	0.13	2010
8	TRANSPLANTATION	25	0.04	2003
10	CELL BIOLOGY	24	0.22	2013

The dual-map overlay of journals (**Figure 5E**) reveals three major citation pathways. Two green citation lines indicate that journals in MEDICINE, MEDICAL, and CLINICAL categories frequently cite those in HEALTH, NURSING, MEDICINE (Citing frequency = 1781) and MOLECULAR, BIOLOGY, GENETICS (Citing frequency = 2901). For instance, journals like *Pediatric Blood Cancer* often cite *Blood* and *NEJM*. A yellow citation line shows frequent citations from MEDICINE, MEDICAL, and IMMUNOLOGY journals to MOLECULAR, BIOLOGY, and GENETICS journals (Citing frequency = 1863), exemplified by journals like *Leukemia* citing *Nature*.

### 3.5 Co-authors, co-cited authors, and leading references

**Tables 6**-**8** highlight the top 10 prolific authors, leading cited authors, and most cited references in pediatric AML prognosis research, with corresponding visualizations in **Figures 6A-C**. Six prolific authors and five leading cited authors are from the United States, underscoring the country’s leading role in this field, as evident across countries, institutions, journals, and authors. Core literature in this domain is identified by citation frequency, with high-impact publications becoming focal points for researchers. Among the top 10 cited authors, Creutzig U, Rubnitz JE, Zwaan CM, and Gamis AS stand out for contributing influential references that have significantly advanced the field. **Figure 6B** and **Table 7** illustrate their extensive collaboration networks. Notably, four of the most cited references are published in *Blood*, reaffirming its leadership in this area (**Table 8**).

**Table 6:** Top 10 authors with the highest documents.

Rank	Author	Countries	Institutions	Documents	Citations	Total link strength
1	Alonzo, Todd a.	US	University of Southern California	69	3225	467
2	Meshinchi, Soheil	US	Fred Hutchinson Cancer Center	67	4240	443
3	Gerbing, Robert b.	US	Children's Oncology Group	53	2373	374
4	Reinhardt, Dirk	DE	German Cancer Research Center	47	2661	339
5	Aplenc, Richard	US	Children's Oncology Group	42	1259	300
6	Hasle, Henrik	DK	Aarhus University	39	1416	284
7	Gamis, Alan s.	US	Children's Mercy Hospital	35	1072	279
8	Kaspers, Gertjanj.	NZ	Princess Maxima Ctr Pediat Oncol	35	1949	204
9	Rubnitz, Jeffrey e.	US	St. Jude Children's Research Hospital	32	2277	163
10	Abrahamsson, Jonas	SE	Queen Silv Childrens Hosp	31	920	250

**Table 7:** Leading cited authors in pediatric AML prognosis research.

Rank	High Cited Authors	Countries	Count	Centrality	Year
1	CREUTZIG U	United States	371	0.13	1999
2	RUBNITZ JE	United States	262	0.04	2003
3	ZWAAN CM	Netherland	212	0.07	2004
4	MESHINCHI S	United States	186	0.15	2002
5	GRIMWADE D	United Kingdom	151	0.14	2000
6	GAMIS AS	United States	142	0.05	2007
7	KASPERS GJL	Netherland	141	0.03	2007
8	UNKNOWN	UNKNOWN	133	0.01	2006
9	GIBSON BES	United States	127	0.04	2007
10	BALGOBIND BV	Netherland	124	0.07	2010

**Table 8:** Leading cited references in pediatric AML prognosis research.

Rank	Count	Title and Authors	DOI
1	113	The molecular landscape of pediatric acute myeloid leukemia reveals recurrent structural alterations and age-specific mutational interactions (Bolouri H etc)	10.1038/nm.4439
2	67	Successes and challenges in the treatment of pediatric acute myeloid leukemia: a retrospective analysis of the AML-BFM trials from 1987 to 2012 (Rasche M etc)	10.1038/s41375-018-0071-7
3	49	Diagnosis and management of AML in adults: 2017 ELN recommendations from an international expert panel (Döhner H etc)	10.1182/blood-2016-08-733196
4	46	Collaborative Efforts Driving Progress in Pediatric Acute Myeloid Leukemia (Zwaan CM etc)	10.1200/JCO.2015.62.8289
5	44	Diagnosis and management of acute myeloid leukemia in children and adolescents: recommendations from an international expert panel (Creutzig U etc)	10.1182/blood-2012-03-362608
6	41	Gemtuzumab Ozogamicin in Children and Adolescents With De Novo Acute Myeloid Leukemia Improves Event-Free Survival by Reducing Relapse Risk: Results From the Randomized Phase III Children’s Oncology Group Trial AAML0531 (Gamis AS etc)	10.1200/JCO.2014.55.3628
7	40	The 2016 revision to the World Health Organization classification of myeloid neoplasms and acute leukemia (Arber DA etc)	10.1182/blood-2016-03-643544
8	40	Minimal Residual Disease-Directed Therapy for Childhood Acute Myeloid Leukemia: Results of the AML02 Multicenter Trial (Rubnitz JE etc)	10.1016/S1470-2045(10)70090-5
9	38	Outcomes in CCG-2961, a Children's Oncology Group Phase 3 Trial for untreated pediatric acute myeloid leukemia: a report from the Children's Oncology Group (Lange BJ etc)	10.1182/blood-2007-04-084293
10	32	Bacillary Angiomatosis in Patients With Cancer: A Pediatric Case Report and a Review of the Literature (Cooper TM etc)	10.1093/jpids/pis085

**Figure 6:**
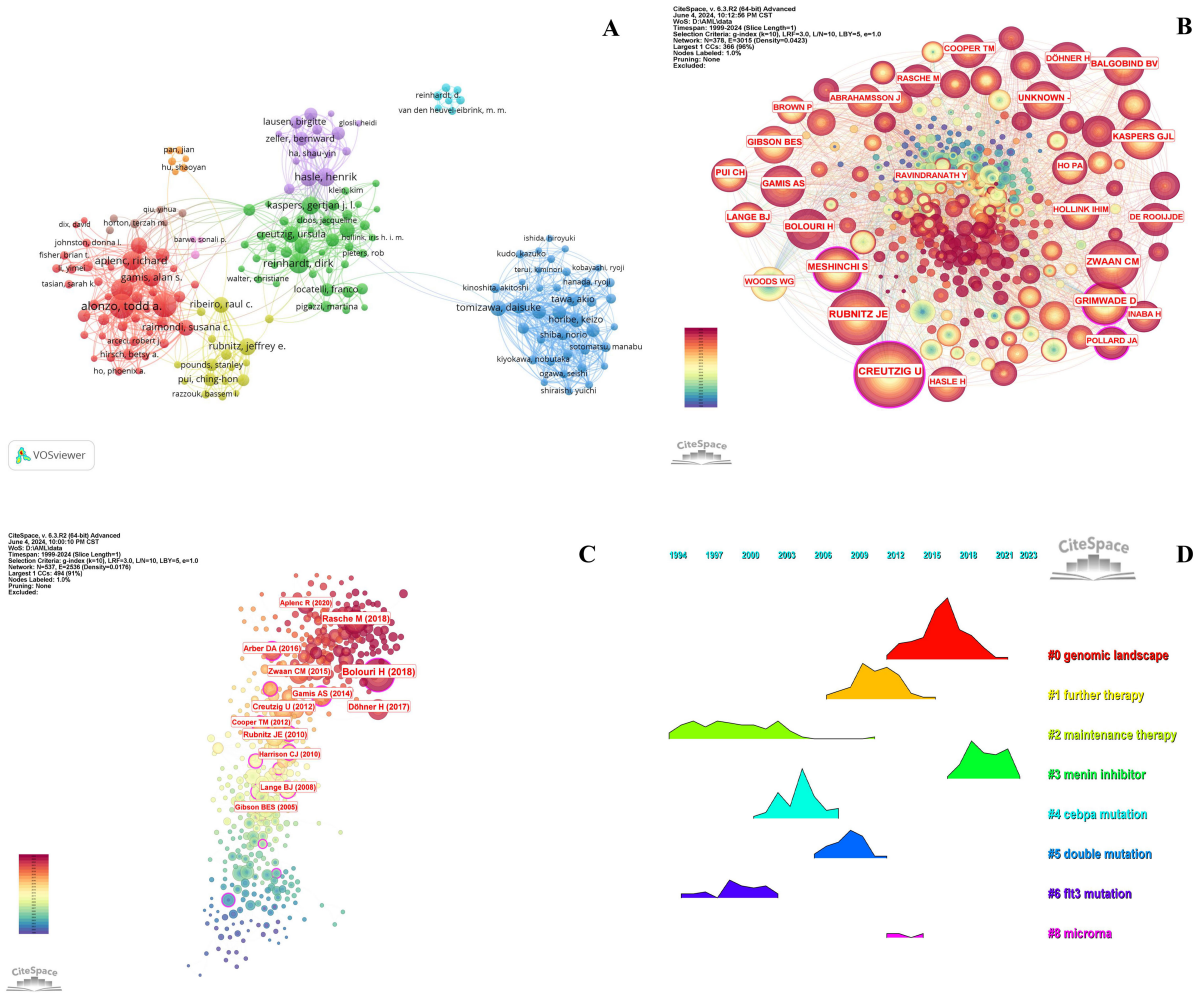
Analysis of authors and references in prognosis-related pediatric AML research. **(A)** The visualization map shows the analysis of authors in prognosis-related pediatric AML research. Each node represents an author, with the node size indicating their number of citations or publications. The lines between the nodes represent collaborative relationships among the authors. **(B)** The cited authors view in pediatric prognosis AML research. The graph illustrates collaborations between cited authors in this field. Each node represents a author, with the node size indicating the citation count. The lines between the nodes represent collaborative relationships between the cited ones. **(C)** The cited references view in pediatric prognosis AML research. The node illustration is similar with **Figure 5B**. **(D)** Ridgeline plot of the cited references in pediatric AML prognosis research. This ridgeline plot visualizes the temporal trends of key cited references in this field. Each colored line represents a distinct research focus, illustrating the evolution of influential topics over time.

The highly cited references fall into three main themes: guidelines and recommendations (e.g., 2017 ELN and 2016 WHO), genomic and epigenetic landscapes (e.g., structural alterations and age-specific mutations), and targeted therapies and clinical trials (e.g., Phase III Children’s Oncology Group Trial AAML0531). These categories reflect pivotal advancements and research priorities in pediatric AML prognosis (**Figure 6C**, **Table 8**). Temporal trends of influential research topics are captured in the ridgeline plot, highlighting key themes such as “genomic landscape” and “further therapy,” which show increasing attention in recent years. Earlier peaks in topics like “menin inhibitor,” “CEBPA mutation,” and “microRNA” indicate prior research focus shifts (**Figure 6D**).

### 3.6 Keywords co-occurrence analysis

**Figure 7:**
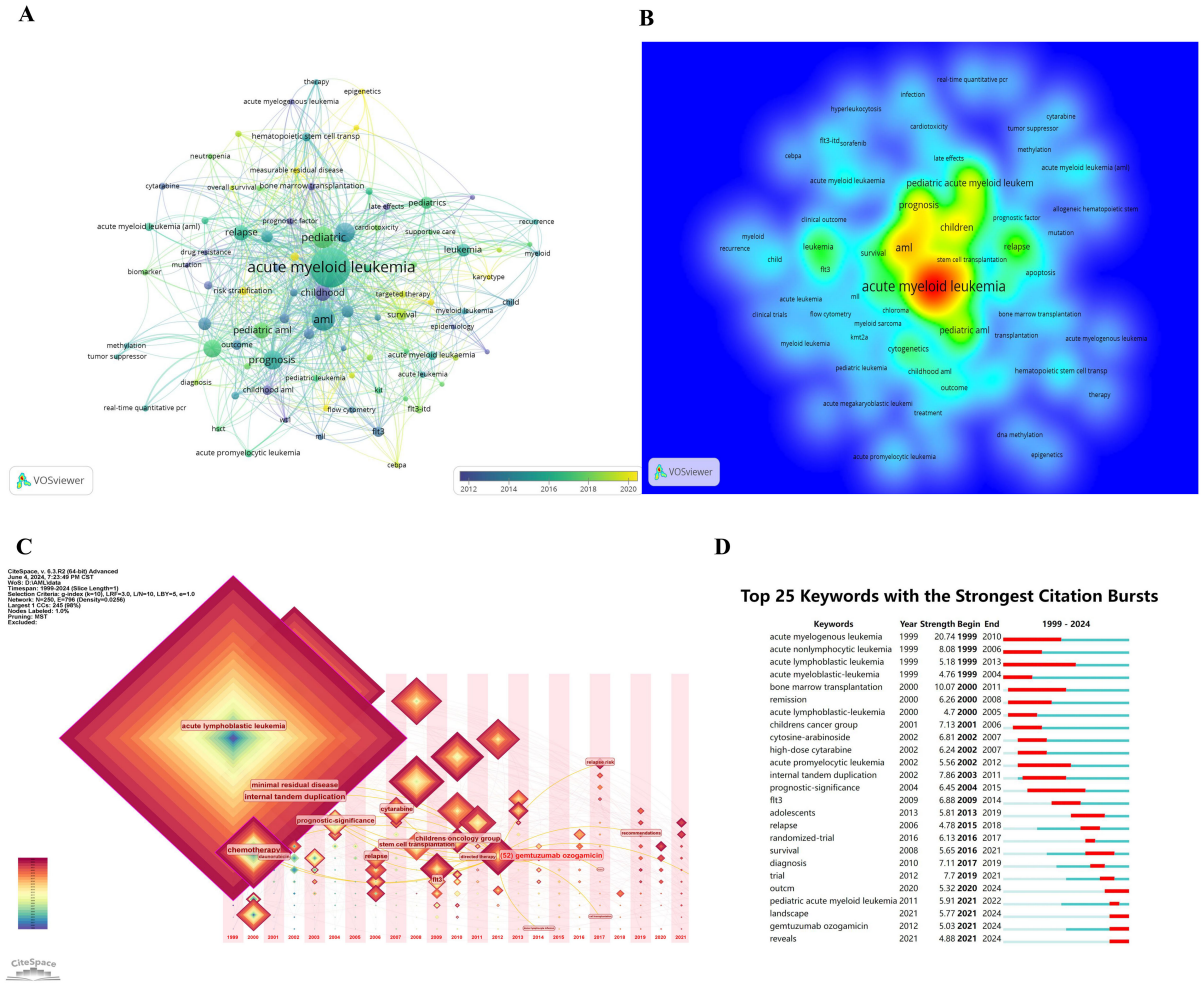
Keywords co-occurrence analysis in prognosis-related pediatric AML research. **(A)** Network visualization of keyword co-occurrence analysis with a minimum of 5 occurrences. Each node represents a keyword, with the node size indicating the number of occurrences. Lines between nodes represent collaborations between keywords; the larger the size of the node, the higher the frequency of the keyword. **(B)** Density visualization of keyword co-occurrence analysis. This visualization displays the density and intensity of research themes in this field. The heatmap is employed to highlight regions of varying study strength, with warmer colors representing areas of higher activity and stronger connections. **(C)** Time zone chart of keyword co-occurrence analysis. This time zone chart visualizes the temporal evolution of key research topics in this field. Each keyword’s prominence is depicted over time, with larger and more central nodes representing higher frequency and significance. **(D)** The 25 keywords with the strongest citation bursts are shown. The blue line represents the time axis, while the red segments indicate the start year, end year, and duration of each burst.

**Figures 7A, B** highlight central themes in pediatric AML prognosis research, including “acute myeloid leukemia,” “pediatric AML,” “prognosis,” “relapse,” “chemotherapy,” “bone marrow transplantation,” “stem cell transplantation,” “immune therapy,” and “survival.” These keywords demonstrate strong interconnections, reflecting their critical role in the research community. The density visualization reveals intense research activity around these themes, with warmer colors indicating high-focus areas. Key genetic factors such as “CEBPA,” “KMT2A,” “WT1,” and “FLT3-ITD,” along with targeted therapies like “sorafenib,” emerge as significant research hotspots, critical to understanding prognostic factors and treatment outcomes.

**Figures 7C, D** illustrate the temporal evolution of research topics. Early focus areas included “chemotherapy” and “acute lymphoblastic leukemia,” while recent trends highlight “minimal residual disease,” “stem cell transplantation,” and “FLT3,” indicating a shift toward advanced therapies and molecular research. **Figure 7D** further identifies the top 25 keywords with the strongest citation bursts, showcasing evolving priorities. Keywords like “acute myelogenous leukemia” (burst period: 1999–2010, strength: 20.74) and “acute lymphoblastic leukemia” (1999–2013, strength: 5.18) demonstrate lasting importance. More recent bursts include “landscape” and “GO,” emerging after 2021, reflecting shifting research interests toward novel directions.

The combined results from **Figures 6D** and **7** underscore the growing prominence of genomics, transcriptomics, epigenomics, targeted therapies, immune therapy, and integrative diagnostic approaches in pediatric AML prognosis research. These findings emphasize the field’s increasing focus on molecular and personalized medicine to improve outcomes.

## 4 Discussion

### 4.1 General information

Research on pediatric AML prognosis has grown substantially over the past 25 years, as reflected by the increasing number of publications and citations. This growth highlights the heightened global focus on improving prognostic outcomes for pediatric AML patients. Our bibliometric analysis, encompassing 924 publications from 1999 to 2023, shows a significant peak in publication and citation activity in 2021, driven by advancements in multi-omics and precision medicine.

Initially, research in this field centered on clinical outcomes and standard chemotherapy regimens (21). Over time, the focus has evolved toward genetic and molecular insights, leading to the discovery of key prognostic markers and novel therapeutic targets (22, 23). This shift underscores the dynamic nature of the field, which continuously adapts to scientific breakthroughs and technological progress (24). High-frequency keywords such as “CEBPA,” “WT1,” and “FLT3-ITD” emphasize the pivotal role of genetic mutations in disease progression and treatment response. Multi-omics approaches—encompassing genomics, transcriptomics, and epigenomics—have further refined prognostic assessments and enabled personalized treatment strategies (23, 25). For example, Pollard and Alonzo demonstrated that adding sorafenib to standard chemotherapy significantly improved event-free survival (EFS) and reduced relapse risk in FLT3/ITD+ pediatric AML patients (26). Similarly, Menin inhibitors demonstrate substantial therapeutic effectiveness in treating KMT2A-rearranged and NPM1-mutated pediatric acute leukemias (27).

Our analysis of contributions from 65 countries highlights a diverse and collaborative research landscape. The United States leads in publication volume, institutional output, journal contributions, categories, and collaborations, underscoring its dominant role in this field (**Tables 1**-**7**, **Figures 3**-**6**). European countries, particularly Germany, have produced high-quality and influential studies, while China, as a developing nation, has shown rapid growth in publication output, reflecting increasing investment in this area (28). Collaboration across countries with varying levels of development and across continents remains vital for fostering innovation and advancing knowledge in pediatric AML prognosis research.

Prominent institutions such as St. Jude Children’s Research Hospital, Erasmus University Rotterdam, and the Children’s Oncology Group (COG) have played instrumental roles in shaping the field. St. Jude leads in publication output, while Erasmus University Rotterdam’s high centrality score reflects its pivotal role in global research networks. COG has contributed groundbreaking clinical trials and comprehensive risk stratification methods, demonstrating the value of multidisciplinary and collaborative approaches. Leading journals, including *Blood* and *Journal of Clinical Oncology*, play a critical role in disseminating high-impact research. These journals’ high impact factors, citation counts, and H-index scores underscore their authority in the field (**Tables 2**, **4**, **8**; **Figures 4**, **6C, D**). Encouraging cross-institutional, cross-journal, and interdisciplinary collaborations could further accelerate knowledge dissemination and foster innovative research.

Pioneering researchers, such as Creutzig U, Rubnitz JE, Zwaan CM, Bolouri H, and Gamis AS, have significantly influenced pediatric AML prognosis research through highly cited publications and seminal references (**Table 8**; **Figures 6B–D**). For instance, Gamis AS demonstrated that GO, a CD33-targeted immunoconjugate, improves three years event-free survival (53.1% vs. 46.9%) and reduces relapse risk (32.8% vs. 41.3%) in children and adolescents (29). Bolouri H’s study on nearly 1,000 participants revealed that somatic structural variants are more prevalent in younger AML patients, with genetic mutations such as GATA2, FLT3, and CBL highlighting the need for age-specific therapeutic strategies (22).

Keyword analysis offers valuable insights into research trends and emerging foci. Terms such as “survival,” “chemotherapy,” “bone marrow transplantation,” “stem cell transplantation,” “immune therapy,” and “relapse” reflect ongoing efforts to improve outcomes. The increasing prominence of genetic and targeted therapies underscores the shift toward precision medicine. Emerging keywords post-2021 reflect the dynamic nature of the field, as researchers continue exploring novel avenues to enhance prognosis and treatment.

This study underscores significant advancements in AML prognosis research, highlighting leading countries, key institutions, prominent journals, major categories, influential contributors, seminal references, and emerging trends. By providing valuable insights into future research directions, this work emphasizes the importance of addressing existing challenges and fostering global collaborations to further advance the field and improve outcomes for pediatric AML prognosis.

In conclusion, this bibliometric analysis provides a comprehensive overview of prognosis-related pediatric AML research, highlighting significant advancements, key contributors, and emerging trends. By identifying influential components, this study offers valuable insights into future research directions. Additionally, this analysis has highlighted several key subtopics within the research on pediatric AML prognosis.

### 4.2 Research progress on pediatric AML prognosis

#### 4.2.1 Genetic insights and risk stratification

Prognostic factors and risk stratification are paramount in guiding treatment decisions and improving outcomes for pediatric AML patients (30). The identification and validation of key genetic and molecular markers have revolutionized our understanding of disease mechanisms and patient prognosis. For instance, FLT3-ITD mutations, especially when combined with WT1 mutations, significantly worsen prognosis and treatment response, necessitating more aggressive treatment protocols (31). Independently, WT1 mutations are associated with poor outcomes and higher relapse rates, highlighting the need for intensified therapeutic strategies (32). The combination of FLT3-ITD and CEBPA mutations further impacts patient survival, with a 3-year overall survival (OS) of only 44%, underscoring the importance of tailored treatment approaches in pediatric AML (33). Conversely, NPM1 mutations, whether accompanied by FLT3-ITD or not, allow for less intensive regimens, reducing treatment-related toxicity while maintaining high survival rates (34, 35).

The discovery of cytogenetic abnormalities has been equally transformative. Chromosomal translocations involving the KMT2A gene are strongly linked to poor outcomes and elevated relapse rates (15). Studies on 11q23/MLL rearrangements reveal significant variations in prognosis depending on specific translocation partners, offering critical insights for risk stratification and treatment planning (36–38). Recent advancements, such as Optical Genome Mapping, have identified novel structural aberrations, enhancing diagnostic accuracy and enabling refined minimal residual disease (MRD) monitoring (39). Additionally, the integration of multi-omics data with machine learning has pinpointed robust molecular markers that predict treatment responses, paving the way for precision medicine in AML (40).

#### 4.2.2 Gene expression profiling

Gene expression profiling is a powerful prognostic tool in pediatric AML (41). Elevated expression of specific genes increases relapse risk, prompting strategies like early stem cell transplantation (42).

Real-time PCR and flow cytometry aid in identifying high-risk patients (43, 44), while next-generation sequencing (NGS) and transcriptome analysis uncover gene fusions such as NPM1-CCDC28A, TRIP12-NPM1, and MLLT10-DNAJC1, enabling precise diagnosis and insights into disease mechanisms (17).

Single-cell sequencing further enhances understanding. scDNA-seq techniques like Phertilizer identify tumor clonal structures via copy number aberrations (45), while scRNA-seq, exemplified by SMART-seq, reveals cellular heterogeneity and gene regulation (46, 47). The NanoString GeoMx Digital Spatial Profiler integrates spatial analysis with single-cell sequencing, mapping tumor microenvironments to identify distinct gene expression patterns critical for understanding disease progression (48). These approaches are vital for advancing tumor biology and targeted therapy.

Epigenetic modifications, especially DNA methylation, predict treatment response and disease progression. DNA methylation clusters linked to cytogenetic abnormalities enhance classification (49), while methylation at bivalent promoters and Polycomb-repressed regions guides therapeutic decisions (50). Specific miRNA profiles also serve as biomarkers for prognosis (51).

Single-cell epigenome sequencing, including scATAC-seq and scChIP-seq, reveals chromatin accessibility and transcription factor binding, advancing gene regulation research. Integrating scRNA-seq and scATAC-seq across treatment time points uncovers transcriptional changes and chromatin dynamics, shedding light on disease progression and treatment resistance (52).

Gene expression profiling drives personalized medicine by identifying genetic abnormalities and molecular signatures, improving risk stratification and enabling precise predictions of disease progression and treatment response, aligning with the rise of precision medicine in pediatric AML.

#### 4.2.3 Multi-omics integration and its influence on prognosis

The integration of molecular genetics, gene expression profiling, and epigenetic modifications has advanced precise and personalized treatment approaches. Patients with favorable genetic profiles can receive fewer intensive therapies, reducing treatment-related toxicity while maintaining high survival rates. In contrast, high-risk patients with adverse genetic markers require more aggressive protocols. As shown in **Figure 7D**, the keyword “landscape” has exhibited the strongest citation burst since 2021, highlighting the growing focus on integrating genomic, transcriptomic, epigenomic, metabolomic, and pharmacogenomic landscapes to provide a comprehensive understanding of pediatric AML pathophysiology (53). The depth and degree of personalization enabled by multi-omics integration are closely interlinked (54), paving the way for more refined therapeutic strategies and improved patient outcomes.

### 4.3 Outcomes in pediatric AML

#### 4.3.1 Remission, induction failure, and transplantation failure

Achieving remission is a crucial objective in pediatric AML treatment. Induction therapy aims to achieve complete remission (CR) by eradicating leukemic cells (55). However, 10-20% of patients fail to reach CR following initial induction therapy (56), often due to factors such as a high initial leukemic burden and adverse genetic profiles.

For patients who achieve CR, sustaining remission and preventing relapse are critical priorities. Allogeneic HSCT is commonly used for high-risk patients in CR1 or those who relapse (57), Nevertheless, transplantation failure and transplant-related mortality remain significant challenge (58, 59). To address these issues, current research focuses on developing less toxic conditioning regimens and improving donor selection to reduce failure rates and enhance overall outcomes.

#### 4.3.2 Relapse and cumulative incidence of relapse

Relapse after remission remains a major challenge in pediatric AML, often resulting in poorer outcomes (60). Key causes of relapse include residual leukemic cells that escape initial treatment, genetic mutations, and chemotherapy resistance. The cumulative incidence of relapse (CIR) varies by risk group, with high-risk patients experiencing significantly higher relapse rates. Studies indicate that approximately 30-40% of pediatric AML patients relapse, highlighting the urgent need for effective post-relapse treatment strategies (60). Minimal residual disease (MRD) is a critical predictor of relapse, with leading references emphasizing its role in guiding therapeutic decisions and improving patient outcomes (8).

#### 4.3.3 Event-free survival and overall survival

Event-free survival (EFS) and overall survival (OS) are critical endpoints for evaluating the efficacy of pediatric AML treatments. EFS measures the duration after treatment during which patients remain free of complications or events, such as relapse or treatment-related mortality. Recent advances have significantly improved EFS rates in pediatric AML, with COG trials reporting 3-year and 5-year EFS rates exceeding 50% for high-risk groups, reflecting notable progress in treatment protocols (61, 62).

OS, defined as the time from diagnosis to death from any cause, has also improved due to enhanced therapeutic strategies. Over the past two decades, 5-year OS rates for pediatric AML have risen to nearly 70% (55). These gains are attributed to better risk stratification, targeted therapies, and advancements in supportive care, all of which contribute to improved patient outcomes. Compared to adult AML, where OS improvements have been slower, pediatric AML has achieved more pronounced advancements, underscoring the effectiveness of tailored pediatric treatment protocols (63).

### 4.4 Clinical trials advancing pediatric AML prognosis

Clinical trials have been pivotal in establishing current treatment standards for pediatric AML, driving advancements in chemotherapy, immunotherapy, targeted therapy, and transplantation. Our bibliometric analysis highlights the importance of clinical trials, as evidenced by the frequent occurrence of keywords such as “treatment,” “clinical trials,” and “stem cell transplantation.” Landmark trials have defined contemporary protocols, such as the COG studies, which consistently demonstrate the efficacy of intensive chemotherapy combined with transplantation, resulting in significant improvements in EFS and OS (61, 64). Similarly, the AML-BFM 98 trial emphasized the effectiveness of risk-adapted therapy, tailoring treatment intensity to individual risk profiles to maximize benefits while minimizing toxicity, reinforcing the value of personalized treatment approaches (65).

Beyond validating existing strategies, clinical trials have paved the way for innovative therapies. The incorporation of FLT3 inhibitors, such as midostaurin and gilteritinib, into treatment protocols has significantly improved survival outcomes for FLT3-ITD-positive patients (66). Additionally, the introduction of GO has enhanced the ability to target CD33-positive leukemic cells, demonstrating efficacy in both frontline and relapsed settings (9, 61, 67). Ongoing trials are further exploring the potential of immunotherapy, including monoclonal antibodies and CAR T-cell therapies, which hold promise for advancing treatment options in pediatric AML (65).

Building on current advancements, new trials are developing strategies to improve outcomes for relapsed and refractory pediatric AML. For example, studies are evaluating the safety and efficacy of Talazoparib, a DNA repair inhibitor, in combination with chemotherapy for relapsed pediatric AML (NCT05101551). Similarly, trials are exploring JK500 cell injections in relapsed/refractory pediatric AML to assess safety and preliminary efficacy (NCT05519384). These efforts, alongside additional studies (NCT03173612, NCT06262438, NCT03164057), are crucial for identifying new therapeutic avenues and optimizing treatment regimens to improve patient outcomes.

### 4.5 Treatment advances and prognosis research in childhood AML

Over the past 25 years, pediatric AML treatment has progressed through evolving strategies, refined risk stratification, and novel therapies, as evidenced by our bibliometric analysis of key trends (**Figure 7**, **Table 9**).

**Table 9:** Evolution of pediatric AML treatment and prognosis over the decades.

Era	Challenges	Keywords	Treatment Development	Prognosis Significance
1990s	Limited chemotherapy effectiveness	"cytosine-arabinoside" “high-dose cytarabine”	“Chemotherapy Protocol”	Initial gains of OS
2000s	Inadequate risk stratification	“internal tandem duplication” “children cancer group”	“Transplantation” “Target Therapy”	Improve EFS
2010s	Relapse and resistance	“CAR-T” “monoclonal antibodies” “MRD”	“Immune Therapy” “Epigenetic Therapy”	Improve life quality Decline CIR
2020s	Personalized treatment integration	“landscape” “multi-omics” “SCS” “AI”	“Comprehensive Therapy” “Precision medicine” “Individualized Therapy”	Improve long-term survival and outcomes

#### 4.5.1 1990s: chemotherapy regimens and initial gains

In the 1990s, pediatric AML treatment relied on intensive chemotherapy with cytarabine and anthracyclines as cornerstone agents. Optimized regimens and improved supportive care significantly enhanced overall survival (OS). Keywords like “cytosine-arabinoside” and “high-dose cytarabine” showed strong citation bursts from 2002 to 2007, reflecting foundational chemotherapy research. Multi-agent chemotherapy during this era notably improved 5-year OS, laying the groundwork for future advancements (68).

#### 4.5.2 2000s: risk stratification and targeted therapies

The early 2000s marked a pivotal shift in pediatric AML treatment, driven by the integration of genetic and molecular insights into risk stratification and the emergence of targeted therapies. Key genetic mutations such as FLT3-ITD, NPM1, and CEBPA were identified, enabling more precise tailoring of treatment protocols to individual patient profiles. Keywords like “internal tandem duplication” and “prognostic-significance” experienced citation bursts during this period, reflecting the growing focus on targeted approaches (69). COG played a critical role in incorporating molecular diagnostics into clinical protocols, with FLT3 inhibitors like midostaurin significantly improving event-free survival (EFS) for high-risk patients (70). This period also saw increased use of bone marrow transplantation for high-risk cases, further enhancing survival outcomes (71).

#### 4.5.3 2010s: progress in immunotherapy and epigenetic therapies

The 2010s marked significant progress in immunotherapy and epigenetic therapies as novel treatment approaches for pediatric AML. Immunotherapy, including monoclonal antibodies and CAR T-cell therapies, enables precise targeting of leukemic cells with fewer side effects than conventional chemotherapy. For example, Anti-CLL1 CAR T-cell therapy targets CLL1 expressed on AML blasts and has shown promise in treating pediatric AML (72). Similarly, PD-1/PD-L1 inhibitors are under investigation for their potential to enhance immune responses against AML cells (73).

Epigenetic therapies, such as azacitidine and decitabine, target DNA methylation and histone modifications, offering new hope for relapsed or refractory AML by reversing aberrant methylation and restoring normal gene expression (74, 75). Additionally, miRNA-based therapies, including mimics and inhibitors, show potential in targeting dysregulated miRNA pathways to improve treatment response and overcome drug resistance (76). Exploring the epigenomic landscape in pediatric AML not only complements traditional therapies but also enhances risk stratification and treatment personalization.

#### 4.5.4 Current challenges in treatment beyond 2020

Since 2020, pediatric AML treatment has increasingly focused on integrating emerging therapies such as CAR-T, GO, and checkpoint inhibitors, alongside a stronger emphasis on precision medicine and individualized care driven by multi-omics insights. Despite these advancements, high relapse and refractory rates, along with challenges in personalized integration, remain significant barriers that impact long-term survival and outcomes. Relapsed or refractory AML is particularly resistant to standard therapies, underscoring the need for innovative treatment strategies (77). Additionally, the toxicity associated with intensive chemotherapy and HSCT, including cardiotoxicity, continues to be a major concern, especially for young patients (78).

### 4.6 Future directions and challenges

Despite significant progress, further exploration is needed to improve pediatric AML prognosis. Novel biomarkers, multi-omics integration, and technologies like machine learning and deep learning offer promising insights into disease mechanisms, personalized therapeutic responses, and prognosis assessment. For example, Pamela S. Becker et al. used a multi-omics computational framework to analyze gene expression profiles and drug sensitivity in 30 AML patients, identifying SMARCA4 as a driver of sensitivity to topoisomerase II inhibitors (40). Support vector machines (SVMs) and advanced statistical tools have identified relapse signatures and predicted therapeutic outcomes, improving risk stratification and enabling personalized treatments, surpassing traditional prognostic models (79, 80). Deep learning applications, such as immune cell segmentation, provide insights into T-cell distribution and its impact on immunotherapy, highlighting immune priming as a strategy to enhance outcomes for patients with low T-cell infiltration (81).

Advanced diagnostic technologies, including NGS, transcriptome sequencing, and SCS, are revolutionizing pediatric AML diagnosis and prognosis. These tools provide comprehensive insights into genetic and molecular drivers, identifying actionable mutations and therapeutic targets. For instance, NGS can detect rare genetic variants associated with treatment resistance, while SCS offers detailed cellular landscape insights to inform the development of targeted therapies (47, 82, 83).

Comprehensive treatment strategies, combining chemotherapy, transplantation, targeted therapy, and immunotherapy, remain critical for improving outcomes in pediatric AML. Notably, CAR T-cell therapy offers significant promise for refractory or relapsed cases (72). Future efforts should prioritize optimizing these combinations to minimize toxicity and enhance long-term survival.

Global disparities in resources present significant challenges to pediatric AML research. High-income countries dominate the field, while low- and middle-income countries face barriers such as limited access to diagnostic tools, fewer clinical trial opportunities, and insufficient research funding. For example, while China is the only developing nation among the top contributors, countries in sub-Saharan Africa, Latin America, and South Asia face similar challenges. Addressing these disparities is critical for fostering a more extensive research landscape. Future efforts should prioritize international collaborations, increase funding for research in low-resource settings, and leverage technologies like telemedicine and mobile diagnostics to bridge gaps. Building infrastructure and providing equitable research opportunities are essential to advancing pediatric AML prognosis research globally.

### 4.7 Limitations

This analysis has several limitations, primarily stemming from its reliance on the Web of Science Core Collection (WOSCC) database, which may exclude regional journals and non-English publications, potentially introducing bias. By focusing solely on English-language articles, the study might overlook valuable research published in other languages. Additionally, while highlighting significant collaborations and contributions from leading countries and institutions, the analysis may miss nuanced contributions from smaller or underrepresented regions, potentially overlooking local peculiarities in global publications. Finally, the emphasis on publication and citation metrics may fail to capture the qualitative aspects of research contributions, such as their practical applicability in clinical settings.

## 5 Conclusion

This bibliometric analysis provides a comprehensive overview of pediatric AML prognosis research over the past 25 years, highlighting key advancements, emerging trends, and transformative innovations. The integration of genetic markers, immunological insights, transcriptomics, and epigenomics has revolutionized risk stratification and treatment strategies, leading to significant improvements in patient outcomes. Advanced tools such as single-cell sequencing (SCS), multi-omics integration, and AI offer valuable support in identifying molecular interaction and novel therapeutic targets. Together with expanded clinical trials and global collaboration, these advancements are crucial for optimizing treatment combinations and improving outcomes for relapsed and refractory cases.

## Data Availability

Publicly available datasets were analyzed in this study. This data can be found here: 10.6084/m9.figshare.28343714.
